# Association of Sleep Quality and Nutritional Status in Middle-Aged and Older Adults: Findings From the WCHAT Study

**DOI:** 10.1093/geroni/igaa057.769

**Published:** 2020-12-16

**Authors:** Wanyu Zhao, Yan Zhang, Birong Dong

**Affiliations:** West China Hospital, Sichuan University, Chengdu, Sichuan, China

## Abstract

Poor sleep quality and malnutrition are two common symptoms which are closely related to the health of middle-aged and older people, but few studies focus on the association between them. In this study, we aimed to identify associations between sleep quality and nutritional status in middle-aged and older adults. A total of 6792 community-dwelling adults aged 50 and older from the baseline of the West China Health and Aging Trend (WCHAT) study were analyzed in the present study. Sleep quality was assessed using the Pittsburgh sleep quality index (PSQI). Scores <=5, 6-10,11-15 and >=16 were categorized as good/mild impaired/moderate impaired/severe impaired sleep quality, respectively. Mini Nutritional Assessment Short Form (MNA-SF) was used to assess the nutritional status and a score<12 was identified as at risk of malnutrition. Logistic regression models were conducted to explore the associations. Of 6792 participants (mean age 62.41 ± 8.26 years, 62.49% women), 1831 (26.96%) had risk of malnutrition. The prevalence of participants with good/mild impaired/moderate impaired/severe impaired sleep quality were 53.72%, 35.54%, 9.61%, and 1.12%, respectively. In the logistic regression model, there were significant association between mild/moderate/severe impaired sleep quality and the presence of risk of malnutrition (OR=1.49, 95% CI=1.32, 1.68; OR=2.15, 95% CI=1.79, 2.59; OR=2.52, 95% CI=1.56, 4.06; all p<0.05) after adjusting for potential confounders. Sleep quality was significantly associated with malnutrition risk with a dosage effect among middle-aged and older adults. Our results highlight the importance of maintaining good sleep quality and nutritional status in middle-aged and older adults.

